# Social Risk Adjustment of Quality Measures for Diabetes and Cardiovascular Disease in a Commercially Insured US Population

**DOI:** 10.1001/jamanetworkopen.2019.0838

**Published:** 2019-03-29

**Authors:** Christina A. Nguyen, Lauren G. Gilstrap, Michael E. Chernew, J. Michael McWilliams, Bruce E. Landon, Mary Beth Landrum

**Affiliations:** 1 Department of Health Care Policy, Harvard Medical School, Boston, Massachusetts; 2 Division of Cardiovascular Medicine, Department of Medicine, Dartmouth Hitchcock Medical Center, Lebanon, New Hampshire; 3 Department of Health Care Policy, The Dartmouth Institute, Dartmouth Medical School, Hanover, New Hampshire; 4 Division of General Internal Medicine, Brigham and Women’s Hospital, Boston, Massachusetts; 5 Division of General Medicine and Primary Care, Beth Israel Deaconess Medical Center, Boston, Massachusetts

## Abstract

**Importance:**

Patients’ social risk factors may be associated with physician group performance on quality measures.

**Objective:**

To examine the association of social risk with change in physician group performance on diabetes and cardiovascular disease (CVD) quality measures in a commercially insured population.

**Design, Setting, and Participants:**

In this cross-sectional study using claims data from 2010 to 2014 from a US national health insurance plan, the performance of 1400 physician groups (physicians billing under the same tax identification number) was estimated. After base adjustments for age and sex, changes in variation across groups and reordering of rankings resulting from additional adjustments for clinical, social, or both clinical and social risk factors were analyzed. In all models, only within-group associations were adjusted to distinguish the association of patients’ social risk factors with outcomes while excluding physician groups’ distinct characteristics that could also change observed performance. Data analysis was conducted between April and July 2018.

**Main Outcomes and Measures:**

Process measures (hemoglobin A_1c_ [HbA_1c_] testing, low-density lipoprotein cholesterol [LDL-C] testing, and statin use), disease control measures (HbA_1c_ and LDL-C level control), and use-based outcome measures (hospitalizations for ambulatory-sensitive conditions) were calculated with base adjustment (age and sex), clinical adjustment, social risk factor adjustment, and both clinical and social adjustments. Quality variance in physician group performance and changes in rankings following these adjustments were measured.

**Results:**

This study identified 1 684 167 enrollees (859 618 [51%] men) aged 18 to 65 years (mean [SD] age, 50 [10.7] years) with diabetes or CVD. Performance rates were high for HbA_1c _and LDL-C level testing (mean ranged from 79.5% to 87.2%) but lower for statin use (54.7% for diabetes cohort and 44.2% for CVD cohort) and disease control measures (57.9% on LDL-C control for diabetes cohort and 40.0% for CVD cohort). On average, only 8.8% of enrollees with diabetes and 1.0% of enrollees with CVD in a group were hospitalized. The addition of clinical and social risk factors to base adjustment reduced variance across physician groups for most measures (percentage change in SD ranged from −13.9% to 1.6%). Although overall agreement between performance scores with base vs full adjustment was high, there was still substantial reordering for some measures. For example, social risk adjustment resulted in reordering for disease control in the diabetes cohort. Of the 1400 physician groups, 330 (23.6%) had performance rankings for HbA_1c_ control that increased or decreased by at least 10 percentile points after adding social risk factors to age and sex. Both clinical and social risk adjustment affected rankings on hospital admissions.

**Conclusions and Relevance:**

Accounting for social risk may be important to mitigate adverse consequences of performance-based payments for physician groups serving socially vulnerable populations.

## Key Points

**Question:**

Is social risk associated with physician group performance on diabetes and cardiovascular disease quality measures?

**Findings:**

In this cross-sectional study of more than 1.6 million enrollees from a large US health insurance plan, adjusting for social risk factors reduced physician group–level variance in performance scores and reordered rankings, particularly for disease control measures for diabetes and use-based outcome measures for both diabetes and cardiovascular disease. Process measure performance did not change significantly following adjustment for social risk factors.

**Meaning:**

Social risk adjustment can affect performance scores for disease control and use-based outcome measures and thus should be considered as a way to mitigate potential unintended consequences of pay-for-performance programs.

## Teaser

This cross-sectional study examines the association of social risk with physician group performance on diabetes and cardiovascular disease quality measures.

## Introduction

The US health care system’s shift toward alternative payment models has reinvigorated interest in adjusting performance measures to account for social risk.^1,2,3,4,5,6,7^ Compared with the traditional fee-for-service model, which rewards volume, alternative payment models commonly reward physicians and physician groups that deliver high-quality care while controlling spending. However, those that serve a disproportionate number of socially disadvantaged patients could be penalized by such a system. Adjusting performance for social risk factors is a way to address this issue.^6,8,9,10,11^ For example, US News Best Hospitals rankings account for social risk using hospital-level percentage of Medicaid patients.^12^

The use of social risk adjustment in payment models is controversial, as raised in reports by the National Academy of Medicine,^13^ US Department of Health and Human Services,^2^ and National Quality Forum.^1^ One argument is that high-risk patients are sicker and have higher health care costs, and the increased difficulty of caring for them is reflected in lower performance scores. Others have argued that because socially disadvantaged patients often receive care from lower-quality institutions, social risk adjustment may obscure true performance or excuse physicians and physician groups that deliver a lower standard of care to disadvantaged patients.^1,2,13,14^ However, risk adjustment methods that separate patient-level risk factors from group-level factors^10,11,13,15^ can be used to adjust for within-physician groups and identify those that provide low-quality care.

Much of the existing work on risk-adjusting performance measures focuses on health plans, hospitals, and Medicare populations. Plans such as those participating in Medicare Advantage already assume population-based risk, and previous research has found a significant association of unadjusted performance with social risk–adjusted performance at the health plan level.^16,17,18,19,20,21^ However, more recent work has found meaningful score changes and substantial reordering for the most penalized hospitals, physician groups, and physicians despite high correlations.^21^ Safety-net hospitals and physicians serving a greater number of dual-eligible Medicare enrollees have been shown to receive more penalties under US Centers for Medicare & Medicaid Services programs.^22,23,24,25,26^ Because most prior studies have focused on associations between unadjusted and adjusted scores, they may miss meaningful changes in observed performance for some physician groups. In addition, the effect of social risk adjustment at the group level and in commercial populations generally has not been well described.

Many population-based payment models have moved away from traditional performance measures, which were dominated by process measures, toward disease control measures, which have been linked to improved life expectancy and physical functioning—outcomes that matter most to patients. These measures can also be advantageous because most clinical outcomes are too rare to be measured reliably at the physician group level (defined as physicians billing under the same tax identification number [TIN]). However, disease control measures may be influenced by nonmedical factors and thus may be particularly sensitive to social risk adjustment.

This study investigated the association of adjusting performance measures for clinical and social risk factors with change in quality measures at the physician group level for a nonelderly, commercially insured population. Adjustment can change absolute scores, rankings, or both. Depending on incentive structures, pay-for-performance programs may take into account any one of these changes. We examined the association of adjustment with changes in scores for disease control measures in addition to other commonly used process measures and use-based outcome measures. We focused on diabetes and cardiovascular disease (CVD), both of which are prevalent and costly chronic diseases in the United States that continue to have suboptimal quality of care.^27,28,29^

## Methods

### Study Overview

This study used data from a large national health insurance plan from January 2010 to December 2014. The member data sets were deidentified, and we obtained institutional review board approval from Harvard University’s Committee on the Use of Human Subjects. The institutional review board did not require informed consent. Data analysis was conducted between April and July 2018. This article is compliant with the Strengthening the Reporting of Observational Studies in Epidemiology (STROBE) reporting guideline for cross-sectional studies.

We constructed 6 diabetes and 4 CVD measures of quality of care: 5 process, 3 disease control, and 2 use-based outcome measures. Adjustment can reduce performance variations across groups (resulting in changes to estimated performance on an absolute scale) and alter groups’ relative rankings. For each measure, we compared the SD, interdecile ranges (the difference between the 90th and 10th percentiles), and changes in rankings across groups resulting from 4 sets of adjustments.

In the base model, we adjusted for age (in 4 age categories: 18-35, 36-45, 46-55, 56-65 years) and sex. In the clinical adjustment model, we added a set of comorbidities (atrial fibrillation, hypertension, chronic obstructive pulmonary disease, heart failure, and chronic kidney disease), coded using the Centers for Medicare & Medicaid Services Chronic Condition Warehouse definitions,^30^ and a comorbidity score based on a DxCG Intelligence Version 5.0.0 (Cotiviti) prediction model. In the social risk–adjustment model, we added zip code–level variables to age and sex (without clinical variables). We used these area-level variables as proxies because individual-level sociodemographic characteristics are generally limited.^1^ Enrollees were linked to zip code–level sociodemographic characteristics from the 2010 US census (percentage of population who are black, Hispanic, and college educated) and the 2010-2014 American Community Survey 5-year estimates (percentage of population below poverty). We assigned enrollees to urban, suburban, and rural classifications based on zip code (eAppendix 1 in the Supplement). Finally, in the fully adjusted model, we included both clinical and social risk covariates in addition to age and sex.

We used medical claims, pharmacy claims, and laboratory results data from 2010 to 2014 from a large national health insurance plan. Our sample included adults aged 18 to 65 years with either diabetes or CVD (based on 2013 Healthcare Effectiveness Data and Information Set eligibility criteria: ≥1 inpatient visit or ≥2 outpatient visits with an *International Classification of Diseases, Ninth Revision (ICD-9) *code for diabetes or CVD who were continuously enrolled in at least 1 calendar (measurement) year between 2010 and 2014. Because pharmacy data were limited to enrollees with pharmacy benefits from the same insurer (47.7% for diabetes cohort and 45.7% for CVD cohort) and laboratory results were available for only some enrollees (52.4% for diabetes cohort and 43.6% for CVD cohort), depending on where they underwent testing, some measures were limited to these subsets.

For each year, we attributed each enrollee to the physician group (defined by TIN) accounting for the plurality of the enrollee’s office visits during that year (eAppendix 2 in the Supplement). Enrollees with the same number of visits to multiple TINs were assigned to the group with the greatest sum of allowed costs.^31^ To ensure sufficient sample size, we restricted our sample to physician groups with at least 40 attributed enrollees with diabetes and at least 40 with CVD, of whom 20 from each cohort had to have laboratory and pharmacy data for every relevant measure.

### Study Variables

We constructed 10 quality measures defined by standard specifications (eAppendix 3 in the Supplement). Diabetes included 3 process measures (hemoglobin A_1c_ [HbA_1c_] testing, low-density lipoprotein cholesterol [LDL-C] testing, and statin use [≥1 filled prescription over the measurement year]), 2 disease control measures (HbA_1c_ level control [<8%; to convert to proportion of total hemoglobin, multiply by 0.01] and LDL-C level control [<100 mg/dL; to convert to millimoles per liter, multiply by 0.0259]), and 1 use-based outcome measure (no hospital admissions for major adverse cardiovascular events [MACE] or diabetes^32^). For CVD, we constructed measures for LDL-C testing, statin use, LDL-C level control, and hospital admissions for MACE. Although the blood cholesterol guidelines switched from being LDL-C–based to risk factor–based in 2013,^33^ we included LDL-C control because it is relevant for most of our data’s time frame (2010-2012 and most of 2013). Measures were dichotomized (1 for adherence, 0 otherwise) and coded so that higher scores indicated better performance.

### Statistical Analysis

We examined the association of enrollees’ clinical and social risk factors with group performance. Specifically, we computed an aggregate performance score for each group as an average of its unadjusted scores on the individual measures and then placed groups into quartiles based on this score.

If disadvantaged enrollees are disproportionately served by low-performing physician groups, then social risk adjustment could mask poor quality. We addressed this problem using a 2-step adjustment process^31^ (eAppendix 4 in the Supplement). First, we fit linear regression models with physician group fixed effects and computed a predicted risk score for each enrollee on each measure based on the relevant covariates. This risk score estimated the likelihood of adherence to the quality metric as a function of patient characteristics, holding group quality constant, as if there were no systematic sorting of enrollees to groups. By basing our adjustments on within-group associations, we captured the associations of patients’ social risk factors while excluding groups’ distinct characteristics associated with observed performance.

Second, to compute group-level performance scores, we estimated mixed-effects logistic regression models that related the actual performance on a given measure to both the risk score computed in step 1 and group random effects. This calculation represents the deviation between observed and expected performance, given a group’s sociodemographic, clinical, and social risk characteristics (depending on the model). Because the mixed-effects logistic model is nonlinear, we standardized across groups using estimated random effects evaluated at the sample mean for all covariates.

Because we identified some systematic differences in observed characteristics between the original cohort and those without pharmacy and/or laboratory data, we standardized the samples across measures. Specifically, we gave each enrollee a weight that was the inverse of the probability that they had laboratory (for disease control measures) or pharmacy (for statin use measures) data. This process gave additional weight to enrollees with data most like those in the full sample and produced samples balanced according to observed characteristics. We estimated probability weights using logistic regression models with availability of data as the dependent variable and the full set of covariates (including all clinical and social risk variables).

We first estimated the SD and interdecile range of the groups’ performances for each of the adjustment types. We then calculated agreement in performance across approaches by computing intraclass correlation coefficients between performance estimates using each type of adjustment and the base adjustment model. Finally, to understand whether some groups would experience meaningful ranking changes, we computed the percentages of physician groups with rankings that increased or decreased at least 10 percentile points after adjustment (for example, a group that moved from the 25th to the 35th percentile).

We tested for differences in patient characteristics across quartiles using trend tests by fitting regression models with quartile as a linear variable. *P *values are 2-sided, and we considered *P* < .05 to be statistically significant. All analyses were performed using Stata statistical software, version 15.1 (StataCorp).

## Results

Our final sample included 1 684 167 unique enrollees (3 069 277 enrollee-years, including 135 485 enrollee-years of diabetes only, 2 339 949 enrollee-years of CVD only, and 593 843 enrollee-years of both) treated by 1400 physician groups. More than half (859 618 [51%]) of enrollees were male, and the mean (SD) age was 50 (10.7) years. There was variation in individuals’ sociodemographic and social risk factors across physician groups (Table 1). For example, physician groups in the top quartile of performance (based on unadjusted mean performance across measures) had a higher percentage of enrollees who were male, aged 56 to 65 years, and had hypertension. In addition, compared with lower-performing groups, those in the highest performance quartile treated more enrollees from zip codes with a greater percentage of white and college-educated individuals and fewer enrollees from zip codes with high rates of poverty.

**Table 1.** Characteristics of Enrollees With Diabetes or CVD^a^

Characteristic	Quartile, %^b^			
	1	2	3	4
Performance score, median (IQR)^c^	67.1 (66.3-68.4)	69.9 (69.5-70.4)	71.6 (71.2-71.9)	74.1 (72.9-74.7)
No. of enrollees, median (IQR)	1095 (455-2322)	1080 (380-2663)	1046 (338-3025)	1010 (449-2621)
Male	50.4	51.7	51.8	52.3
Age, y				
18-35	8.5	7.6	6.9	6.1
36-45	18.3	17.9	17.1	16.3
46-55	34.7	34.9	35.4	35.3
56-65	37.1	38.8	39.4	41.8
Comorbidities				
Atrial fibrillation	1.1	1.1	1.1	1.1
Hypertension	35.1	35.2	36.8	38.0
Chronic obstructive pulmonary disease	2.0	2.0	2.0	1.7
Heart failure	1.8	1.5	1.5	1.4
Chronic kidney disease	2.4	2.3	2.2	2.3
Zip code−level characteristics				
White	66.1	72.9	74.0	74.6
Black	11.9	11.1	10.3	10.8
Hispanic	18.5	13.2	12.6	14.0
College educated	26.2	29.2	30.2	32.1
Below poverty	13.7	12.5	11.7	11.0
Urban	56.0	47.3	44.3	60.1
Suburban	42.3	51.0	52.5	38.6
Rural	0.8	1.3	1.1	1.0

Abbreviations: CVD, cardiovascular disease; IQR, interquartile range.

^a^ Median percentages of unique enrollee-years aggregated to the physician group level.

^b^ Quartiles 1 to 4 are ordered from low to high quality. Trend tests showed significant differences across quartiles for all characteristics (*P* < .05).

^c^ Performance score computed as the mean performance on all unadjusted quality measures.

Mean performance rates were high for measures of testing in both cohorts (mean ranged from 79.5% to 87.2%) (Table 2). Mean performance was lower—and variation across groups was higher—for statin use (54.7% for diabetes cohort and 44.2% for CVD cohort) and disease control measures (57.9% on LDL-C control for diabetes cohort and 40.0% for CVD cohort). For example, the mean (interdecile range) for HbA_1c_ control was 69.4% (62.5%-75.7%). Only 1.0% of CVD enrollees had an admission for MACE, with little variation across groups. Hospitalization for the diabetes cohort for MACE was higher (8.8%) and more variable (interdecile range, 88.3%-93.6%) across groups in the diabetes cohort.

**Table 2.** Distribution of Unadjusted Physician Group Performance on Diabetes and Cardiovascular Disease Measures^a^

Measure	Condition	Performance Score, Mean (SD)	Coefficient of Variation (Interdecile Range)
Process			
Hemoglobin A_1c_ testing	Diabetes	87.2 (4.4)	0.05 (81.6-92.0)
Low-density lipoprotein cholesterol testing	Diabetes	83.6 (5.6)	0.07 (76.4-89.9)
Low-density lipoprotein cholesterol testing	Cardiovascular disease	79.5 (5.3)	0.07 (72.9-85.9)
Any statin use	Diabetes	54.7 (5.8)	0.11 (47.2-61.8)
Any statin use	Cardiovascular disease	44.2 (7.4)	0.17 (34.8-53.1)
Disease control			
Hemoglobin A_1c_ level control (<8%)	Diabetes	69.4 (5.3)	0.08 (62.5-75.7)
Low-density lipoprotein cholesterol level control (<100 mg/dL)	Diabetes	57.9 (4.4)	0.08 (52.3-63.5)
Low-density lipoprotein cholesterol level control (<100 mg/dL)	Cardiovascular disease	40.0 (4.7)	0.12 (34.4-45.5)
Use-based outcome			
Hospital admissions	Diabetes	91.2 (2.3)	0.03 (88.3-93.6)
Hospital admissions	Cardiovascular disease	99.0 (0.2)	0.01 (98.7-99.3)

SI conversion factors: To convert hemoglobin A_1c _to proportion of total hemoglobin, multiply by 0.01; to convert low-density lipoprotein cholesterol to millimoles per liter, multiply by 0.0259.

^a^ Based on the percentage of patients adhering to a quality measure in a physician group.

Most clinical and social risk factors were significantly associated with all quality measures at the individual level (eTables 1 and 5-7 in the Supplement). The explanatory powers of the individual covariates were similar across adjustment models, and their directions were consistent with prior research. For most measures, younger age, male sex, and percentage of college-educated individuals in the zip code were positively associated with higher performance. Most comorbidities, more rural geography, and percentage black and below poverty were negatively associated with performance. The DxCG prediction model composite score was negatively associated with performance on process measures and hospitalizations for ambulatory-sensitive conditions but positively associated with disease control. The percentage of Hispanic individuals in an enrollee’s zip code had inconsistent associations.

### Variation Across Physician Groups After Adjustment

Variance across physician groups decreased after full adjustment for most measures (percentage change in SD ranged from −13.9% for HbA_1c_ level control in the diabetes cohort to 1.6% for hospital admission in the CVD cohort) (eTable 2 in the Supplement). Adjustment for social risk factors was associated with variation across groups in disease control and hospital admissions in the diabetes cohort (Figure). For example, the interdecile range for performance on HbA_1c_ level control in the diabetes cohort decreased from 12.8 percentage points with base adjustment to 10.6 percentage points after full adjustment. In contrast, the interdecile range in HbA_1c_ testing in the diabetes cohort changed minimally. Variation across groups in admissions for the diabetes cohort was sensitive to both clinical and social risk adjustment.

**Figure.**
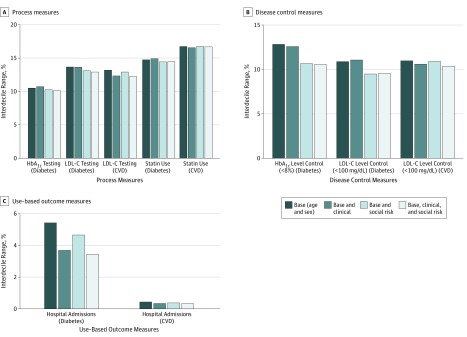
Performance on Diabetes and Cardiovascular Disease (CVD) Measures by 1400 Physician Groups After Adjustment HbA_1c_ indicates hemoglobin A_1c_; LDL-C, low-density lipoprotein cholesterol. To convert HbA_1c _to proportion of total hemoglobin, multiply by 0.01; to convert LDL-C level to millimoles per liter, multiply by 0.0259.

### Performance Scores and Rankings

Overall agreement between group-level performance scores with base adjustment vs adjustment for clinical, social risk, or both factors was high (eTable 3 in the Supplement). However, correlations between unadjusted and adjusted performance were weaker for admission measures (comparing full with base adjustment, intraclass correlation coefficient for diabetes was 0.84; intraclass correlation coefficient for CVD was 0.76). Performance on these use-based outcomes was most heavily affected by adjustment for clinical variables.

Overall agreement between unadjusted and adjusted scores can be high despite important score changes and rankings for some physician groups, particularly if they treat a disproportionate number of enrollees with greater clinical and social risk. We found performance ranking increases or decreases of at least 10 percentile points after social risk adjustment in 330 physician groups (23.6%) for HbA_1c_ level control and in 129 (9.2%) for LDL-C level control for diabetes (Table 3 and eTable 4 in the Supplement). Both clinical and social risk factors reordered rankings for admissions measures, although the effect of clinical variables was larger.

**Table 3.** Movement by 10 Percentiles or More After Adjustment Among 1400 Physician Groups

Measure	Condition	Physician Groups, No. (%)		
		Clinical Adjustment	Social Risk Adjustment	Full Adjustment
Process				
Hemoglobin A_1c_ testing	Diabetes	127 (9.1)	2 (0.1)	123 (8.8)
Low-density lipoprotein cholesterol testing	Diabetes	97 (6.9)	6 (0.4)	110 (7.9)
Low-density lipoprotein cholesterol testing	Cardiovascular disease	112 (8.0)	4 (0.3)	89 (6.4)
Any statin use	Diabetes	111 (7.9)	21 (1.5)	125 (8.9)
Any statin use	Cardiovascular disease	143 (10.2)	0	100 (7.1)
Disease control				
Hemoglobin A_1c_ level control (<8%)	Diabetes	0	330 (23.6)	337 (24.1)
Low-density lipoprotein cholesterol level control (<100 mg/dL)	Diabetes	12 (0.9)	129 (9.2)	142 (10.1)
Low-density lipoprotein cholesterol level control (<100 mg/dL)	Cardiovascular disease	161 (11.5)	29 (2.1)	134 (9.6)
Use-based outcome				
Hospital admissions	Diabetes	466 (33.3)	275 (19.6)	537 (38.4)
Hospital admissions	Cardiovascular disease	512 (36.6)	305 (21.8)	509 (36.4)

SI conversion factors: To convert hemoglobin A_1c _to proportion of total hemoglobin, multiply by 0.01; to convert low-density lipoprotein cholesterol to millimoles per liter, multiply by 0.0259.

## Discussion

In this study of commercially insured enrollees with diabetes or CVD, we found that adherence to standard quality measures was associated with patients’ social risk factors. Although risk variables were significant at the individual patient level, our results show similar rankings in group-level performance after case-mix adjustment, consistent with previous studies focusing on health plans^16,18^ and hospitals.^34,35^ However, high agreement between scores with and without social risk adjustment should not be interpreted as evidence that such adjustments altered performance scores minimally, as we also found that adjustment resulted in changes in performance rankings for a subset of physician groups as well as a substantial reduction in performance variation on some measures that did not result in reordering. Thus, payment programs relying on these measures with limited adjustments could penalize groups serving socially disadvantaged patients.

Moreover, the method of risk adjustment is important. Traditional regression approaches to social risk adjustment could mistakenly attribute low quality to social risk factors, masking truly poor performance.^1,2,13^ This would result in a lower standard of care for patients with greater clinical and social risk. However, two 2018 articles by Roberts et al^11,15^ demonstrated how risk adjustment can be based on within-group associations between patient characteristics and quality outcomes, thereby excluding physician groups’ distinct contributions. Thus, it is important, as we did in this study, to estimate the association of social risk factors with quality measures within groups because it adjusts for this sorting and thus avoids this bias. Adjusting for within-group associations differentiated between the association of patients’ social risk factors with quality measures and between-group differences in quality.

Overall, there was greater variance reduction and rank reordering from social risk adjustment for disease control and use-based outcome measures than for process measures. This may be a result of process measures being topped out, in that most physician groups achieve high scores, or it could be because patient outcomes may be more vulnerable to other, unmeasured confounders than process measures. In both cohorts, we observed little variation in statin use, a measure with lower and more variable performance across groups that has not been frequently used in alternative payment models. Disease control measures have become increasingly important in quality measurement systems as proxies for less common outcomes, but they are less controllable than process measures, which are easier to achieve. Thus, the association of social risk adjustment with disease control measures is important to consider when developing programs that evaluate quality performance. In this study, we considered disease control measures that can be determined using standard laboratory claims data. Other important disease control measures, such as blood pressure control, would be more challenging and costly to evaluate, as they would require medical record review.

For some measures, including hospitalizations for ambulatory-sensitive conditions, clinical variables had a greater association with outcome on variation and ranking across physician groups. However, the addition of social risk factors changed rankings in disease control for diabetes even after adjustment for clinical factors, suggesting that the associations of social risk with disease control are not entirely manifested through poor clinical health among socially disadvantaged enrollees. For example, blood glucose levels are largely determined by diet, exercise, and lifestyle (including the ability to adhere to medications and treatment)—all activities associated with socioeconomic factors. In contrast, rankings were reordered less following social risk adjustment for LDL-C performance. This may be because statins are quite effective, and even those patients with poor lifestyle habits can achieve good LDL-C levels. Importantly, our findings refute the assertion that risk adjustment with minimal changes to variance implies a corresponding lack of change in quality ranking.^13^

The association of social risk adjustment with performance has different implications for physician groups and their patients under various pay-for-performance schemes. If the size of the bonus or penalty depends on the score’s deviation from a mean (eg, Accountable Care Organization programs, Value-Based Payment Modifier), variance is important even if there is no reordering. On the other hand, if rewards and penalties are based on rankings, variance reduction without reordering would not matter. We found evidence of sizable ranking changes among a meaningful minority of physician groups as well as variance reduction in disease control measures, which would affect bonuses and penalties in pay-for-performance programs that base scores on either deviations from a mean or rankings. Both variance reduction and rank reordering are important for public reporting.

Risk adjustment is not the only factor contributing to the validity and reliability of performance measures. Measurement error and statistical noise in small samples are well-known factors, and rankings are particularly sensitive to these issues.^36^ Differing methods for aggregating individual measures into composite scores can also reorder rankings.^37,38^

### Limitations

Our study has limitations. First, our data were not sufficient to examine the association of individual-level risk factor adjustments with changes in quality measures. Because collection of these data continues to be limited,^1^ area-level social risk factors are being more widely considered in policy and serve as proxies for both individual-level sociodemographic characteristics and characterizations of patients’ communities. It may be more practical to use area-level factors and avoid adding to the burden of data collection. Although recent work has shown that aggregate proxies at the 9-digit zip code level can provide more precision,^39^ we unfortunately only had access to 5-digit zip codes in our data. Nevertheless, adjustment for individual-level data would likely produce even larger variance reduction and reordering, and our analysis of area-level factors highlights the importance of improving the collection and availability of data on individual-level risk factors in the future.^13^ Second, with our data, we could only examine use-based outcomes and not other clinical outcomes. The normative interpretation of use-based outcomes can be ambiguous because some social risk factors may be associated with factors that may predict lower demand and use of care (eg, insurance coverage); for example, increased admissions may not always signal poorer quality of care, particularly for chronic diseases. Third, we found moderate but important differences in this younger, commercially insured population. Adjustment for social risk factors in more diverse populations would likely be associated with larger changes to variance and rankings.

## Conclusions

As alternative payment models increasingly rely on standard quality measures of physician and physician group performance to define and reward high-quality care, our findings suggest that inadequate risk adjustment could counterproductively reduce payments to groups whose patient populations would benefit most from additional resources, including social interventions. Physician group rankings on disease control measures are among those most altered by social risk adjustment, particularly for diabetes. Use of these measures to determine group payment without adjustment for social risk factors could lead to fewer resources for physicians caring for populations with greater clinical and social risk and exacerbate disparities in care.

## Notes

Supplement. **eAppendix 1**. Urban/Suburban/Rural Zip Code Classification **eAppendix 2.** Enrollee Attribution to TIN **eAppendix 3.** Quality Metric Coding **eAppendix 4.** Two-Step Adjustment Process **eTable 1. **R-squared (%) for Predicting Physician Performance **eTable 2. **Standard Deviation in Percentage Points (% Change Compared to Base) **eTable 3. **Intraclass Correlation Coefficients Between Physician Group Performance Rankings With Base Versus Other Adjustments **eTable 4.** Number (%) of Physician Groups Moving 5 Percentiles or More After Adjustment (N = 1400) **eTable 5. **Regression Results for Process Measures **eTable 6. **Regression Results for Disease Control Measures **eTable 7. **Regression Results for Use-Based Outcome Measures **eReferences**
