# Omission in Supplement

**DOI:** 10.1001/jamanetworkopen.2019.4723

**Published:** 2019-05-10

In the Original Investigation titled “Social Risk Adjustment of Quality Measures for Diabetes and Cardiovascular Disease in a Commercially Insured US Population,”^1^ published March 29, 2019, the following statement should have been included in eAppendix 3 in the Supplement: “Utilization-Based Outcome Measures: (1) any hospitalization for diabetes, including observational stays; (2) any hospitalization for major adverse cardiovascular events, including observational stays; (3) emergency department visit for diabetes (patient was not admitted); and (4) emergency department visit for major adverse cardiovascular events (patient was not admitted).” The final sentence of eAppendix 3 in the Supplement should have read: “Admissions/emergency department visits for diabetes were determined using the Agency for HealthCare Research and Quality’s (AHRQ) prevention quality indicators (PQI 1, 3, 14 and 16) for diabetes.” This article has been corrected.^1^
