# Supplementary material for: Social Risk Adjustment of Quality Measures for Diabetes and Cardiovascular Disease in a Commercially Insured US Population
Source: JAMA Netw Open. 2019 Mar 29;2(3):e190838. doi: 10.1001/jamanetworkopen.2019.0838 (PMC6450315; doi:10.1001/jamanetworkopen.2019.0838)
Supplement: Supplement. — eAppendix 1. Urban/Suburban/Rural Zip Code Classification eAppendix 2. Enrollee Attribution to TIN eAppendix 3. Quality Metric Coding eAppendix 4. Two-Step Adjustment Process eTable 1. R-squared (%) for Predicting Physician Performance eTable 2. Standard Deviation in Percentage Points (% Change Compared to Base) eTable 3. Intraclass Correlation Coefficients Between Physician Group Performance Rankings With Base Versus Other Adjustments eTable 4. Number (%) of Physician Groups Moving 5 Percentiles or More After Adjustment (N = 1400) eTable 5. Regression Results for Process Measures eTable 6. Regression Results for Disease Control Measures eTable 7. Regression Results for Use-Based Outcome Measures eReferences [file jamanetwopen-2-e190838-s001.pdf]

Supplementary Online Content

Nguyen CA, Gilstrap LG, Chernew ME, McWilliams JM, Landon BE, Landrum MB. Social risk adjustment of quality measures for diabetes and cardiovascular disease in a commercially insured US population. *JAMA Netw Open.* 2019;2(3):e190838. doi:10.1001/jamanetworkopen.2018.0838

eAppendix 1. Urban/Suburban/Rural Zip Code Classification

eAppendix 2. Enrollee Attribution to TIN

eAppendix 3. Quality Metric Coding

eAppendix 4. Two-Step Adjustment Process

eTable 1. R-squared (%) for Predicting Physician Performance

eTable 2. Standard Deviation in Percentage Points (% Change Compared to Base)

eTable 3. Intraclass Correlation Coefficients Between Physician Group Performance Rankings With Base Versus Other Adjustments

eTable 4. Number (%) of Physician Groups Moving 5 Percentiles or More After Adjustment (N = 1400)

eTable 5. Regression Results for Process Measures

eTable 6. Regression Results for Disease Control Measures

eTable 7. Regression Results for Use-Based Outcome Measures

eReferences

This supplementary material has been provided by the authors to give readers additional information about their work.

© 2019 Nguyen CA et al. *JAMA Network Open.*

eAppendix 1. Urban/Suburban/Rural Zip Code Classification

Building on methods from a previous study and corresponding to geographical distinctions made in the reporting of the Behavioral Risk Factor Surveillance Survey,[1] we classified ZCTAs into 3 categories: urban, suburban, and rural. Using the 2010 census metro and micro delineation file, we identified the principal city in each core based statistical area (CBSA) then employed a crosswalk to flag as urban the ZCTAs located in each principal city. Using the census urban area relationship file, we flagged as rural all ZCTAs determined to be not in urban areas. All ZCTAs not flagged as urban or rural were classified as suburban; we reclassified 41 urban ZCTAs with fewer than 20 residents per square mile as suburban ZCTAs.[2]

© 2019 Nguyen CA et al. *JAMA Network Open.*

eAppendix 2. Enrollee Attribution to TIN

In each year, we attributed each enrollee to the provider group (defined by Tax Identification Number) accounting for the plurality of the enrollee's office visits during the year: current procedural terminology [CPT] codes 99201-99215, 99241-99245, G0402, G0438, G0438, with specialty codes for family medicine [08], internal medicine [11], geriatric medicine [38], general provider organization [01] or endocrinology [46]. Enrollees with the same number of visits to more than one TIN were assigned to the TIN with the greater sum of allowed costs. TINs represent provider groups ranging from small practices to health systems.

© 2019 Nguyen CA et al. *JAMA Network Open.*

eAppendix 3. Quality Metric Coding

Testing Measures

Metric	Numerator	Denominator
**% with HbA_{1c} Test**	Eligible enrollees with ≥1 test for HbA_{1c} in claims during the measurement year	All eligible enrollees [a]
**% with LDL-C Test**	Eligible enrollees with ≥1 test for LDL-C in claims during the measurement year	All eligible enrollees [a]

[a] Eligible Enrollees: enrollees with a diagnosis of diabetes or CVD (≥1 inpatient or ≥2 outpatient claims for diabetes or CVD during the measurement year) and attributed to a TIN with ≥40 attributed enrollees with diabetes and ≥40 with CVD.

Disease Control Measures

Metric	Numerator	Denominator
**% with HbA_{1c} <8%**	Eligible enrollees* whose first HbA_{1c} value in the measurement year was ≥8%	Eligible enrollees [b] with ≥1 laboratory test for HbA_{1c} (from the laboratory file) during the measurement year
**% with LDL-C <100mg/dL**	Eligible enrollees* whose first LDL-C value in the measurement year was ≥100mg/dL	Eligible enrollees [b] with ≥1 laboratory test for LDL-C (from the laboratory file) during the measurement year

[b] Eligible Enrollees: enrollees with a diagnosis of diabetes or CVD (≥1 inpatient or ≥2 outpatient claims for diabetes or CVD during the measurement year), with laboratory data, and attributed to a TIN with ≥40 attributed enrollees with diabetes and ≥40 with CVD.

Drug-Use Measures

Metric	Numerator	Denominator
**% with use of any statin**	Eligible enrollees [c] with ≥1 fill of any statin. Statin use identified in the pharmacy file using National Drug Codes (NDC) [d] during the measurement year	All eligible enrollees [c]

[c] Eligible Enrollees: enrollees with a diagnosis of diabetes or CVD (≥1 inpatient or ≥2 outpatient claims for diabetes or CVD during the measurement year), with pharmacy data, and attributed to a TIN with ≥40 attributed enrollees with diabetes and ≥40 with CVD.

[d] Statin NDC codes obtained from HEDIS 2016 and are available at: http://www.ncqa.org/hedis-quality-measurement/hedis-measures/hedis-2016/hedis-2016-ndc-license/hedis-2016-final-ndc-lists (Access Date May 21, 2018).

© 2019 Nguyen CA et al. *JAMA Network Open.*

Major Adverse Cardiovascular Events (MACE) Utilization-Based Outcome Measures

Acute Coronary Syndrome: 410.x

Angina: 411.1, 411.8x, 413.x

Cerebrovascular accident/stroke: 430-432, 433-436

Malignant dysrhythmia: 427.1, 427.4, 427.41-427.42, 427.5

Sudden Cardiac Death: 798.1, 798.2

Coronary Revascularization CPT: 33510-33519, 33520-33523, 33530-33536, 92973-92984, 92995-92998

Coronary Revascularization HCPCS: S2205-S2209, G0290, G0291

Admissions for diabetes were determined using the Agency for HealthCare Research and Quality's (AHRQ) prevention quality indicators (PQI 1, 3, 14 and 16) for diabetes.[3]

© 2019 Nguyen CA et al. *JAMA Network Open.*

eAppendix 4. Two-Step Adjustment Process

First, we computed a predicted performance score for each enrollee on each measure based on the relevant covariates but taking out the effects of the provider group. To do this, we fit linear regression models at the enrollee-year level with fixed effects for provider group (pooled across all years). Using estimated coefficients from these models, we computed a enrollee-year-level predicted performance for each measure, equal to the predicted performance estimated from only the coefficients on the enrollee characteristics (i.e., not including the coefficients on the group fixed effects in the prediction). This predicted performance is what we would expect if we did not have any systematic sorting of enrollees to provider groups.

Second, to compute group-level performance scores, we estimated mixed effects logit regression models that related the actual performance on a measure to the predicted performance computed in step 1 and group random effects. This represents the deviation between observed and expected performance given a group's sociodemographic, clinical, and social risk (depending on the model). Because the mixed effects logit is non-linear, we standardize across groups using estimated random effects evaluated at the sample mean for all covariates.

© 2019 Nguyen CA et al. *JAMA Network Open.*

eTable 1. R-squared (%) for Predicting Physician Performance

Measure Type	Measure	Condition	Unadjusted	Base	Clinical	Social Risk	Full
Process	HbA_{1c} testing	Diabetes	2.33	2.82	4.82	2.85	4.88
	LDL-C testing	Diabetes	2.61	3.85	6.12	3.91	6.21
	LDL-C testing	CVD	1.51	2.51	4.32	2.52	4.35
	Any statin use	Diabetes	1.9	9.09	10.42	9.12	10.49
	Any statin use	CVD	1.93	9.57	11.1	9.57	11.12
Disease Control	HbA_{1c} level control (<8%)	Diabetes	1.81	3.26	3.43	3.61	3.75
	LDL-C level control (<100 mg/dL)	Diabetes	1.41	3.32	3.58	3.44	3.73
	LDL-C level control (<100 mg/dL)	CVD	0.89	2.9	4.21	2.91	4.23
Outcome	Hospital admissions	Diabetes	1.04	1.11	13.75	1.35	13.87
	Hospital admissions	CVD	0.15	0.36	8.58	0.39	8.58

© 2019 Nguyen CA et al. *JAMA Network Open.*

eTable 2. Standard Deviation in Percentage Points (% Change Compared to Base)

Measure Type	Measure	Condition	Base	Clinical	Social Risk	Full
Process	HbA_{1c} testing	Diabetes	4.45	4.33 (-2.8)	4.40 (-1.2)	4.22 (-5.4)
	LDL-C testing	Diabetes	4.58	4.40 (-4.1)	4.51 (-1.6)	4.27 (-7.4)
	LDL-C testing	CVD	3.58	3.47 (-3.2)	3.54 (-1.3)	3.41 (-5.2)
	Any statin use	Diabetes	2.99	3.01 (0.8)	2.96 (-0.9)	2.94 (-1.5)
	Any statin use	CVD	3.12	3.12 (-0.1)	3.13 (0.2)	3.10 (-0.5)
Disease Control	HbA_{1c} level control (<8%)	Diabetes	3.08	3.06 (-0.6)	2.71 (-13.9)	2.71 (-13.9)
	LDL-C level control (<100 mg/dL)	Diabetes	2.31	2.35 (1.6)	2.11 (-9.8)	2.11 (-9.9)
	LDL-C level control (<100 mg/dL)	CVD	2.18	2.12 (-2.8)	2.19 (0.4)	2.10 (-3.7)
Outcome	Hospital admissions	Diabetes	3.46	3.45 (-0.1)	3.14 (-10.0)	3.34 (-3.7)
	Hospital admissions	CVD	3.13	3.19 (2.1)	2.85 (-9.6)	3.18 (1.6)

© 2019 Nguyen CA et al. *JAMA Network Open.*

eTable 3. Intraclass Correlation Coefficients Between Physician Group Performance Rankings With Base Versus Other Adjustments

Measure Type	Measure	Condition	Mean	Interdecile Range (10th-90th)	Clinical	Social Risk	Full
Process	HbA_{1c} testing	Diabetes	87.2	81.6-92.0	0.98	1.0	0.98
	LDL-C testing	Diabetes	83.6	76.4-89.9	0.98	1.0	0.98
	LDL-C testing	CVD	79.5	72.9-85.9	0.98	1.0	0.99
	Any statin use	Diabetes	54.7	47.2-61.8	0.99	1.0	0.98
	Any statin use	CVD	44.2	34.8-53.1	0.98	1.0	0.99
Disease Control	HbA_{1c} level control (<8%)	Diabetes	69.4	62.5-75.7	1.0	0.96	0.96
	LDL-C control (<100 mg/dL)	Diabetes	57.9	52.3-63.5	1.0	0.98	0.98
	LDL-C control (<100 mg/dL)	CVD	40.0	34.4-45.5	0.98	1.0	0.98
Outcome	Hospital admissions	Diabetes	91.2	88.3-93.6	0.88	0.96	0.84
	Hospital admissions	CVD	99.0	98.7-99.3	0.76	0.95	0.76

Correlations were all statistically significant at the p<0.001 level.

© 2019 Nguyen CA et al. *JAMA Network Open.*

eTable 4. Number (%) of Physician Groups Moving 5 Percentiles or More After Adjustment (N = 1400)

Measure Type	Measure	Condition	Clinical	Social Risk	Full
Process	HbA_{1c} testing	Diabetes	493 (35.2)	101 (7.2)	484 (34.6)
	LDL-C testing	Diabetes	423 (30.2)	142 (10.1)	450 (32.1)
	LDL-C testing	CVD	419 (29.9)	82 (5.9)	377 (26.9)
	Any statin use	Diabetes	416 (29.7)	110 (7.9)	429 (30.6)
	Any statin use	CVD	501 (35.8)	11 (0.8)	428 (30.6)
Disease Control	HbA_{1c} level control (<8%)	Diabetes	8 (0.6)	707 (50.5)	714 (51.0)
	LDL-C level control (<100 mg/dL)	Diabetes	112 (8.0)	432 (30.9)	433 (30.9)
	LDL-C level control (<100 mg/dL)	CVD	512 (36.6)	197 (14.1)	486 (34.7)
Outcome	Hospital admissions	Diabetes	848 (60.6)	613 (43.8)	903 (64.5)
	Hospital admissions	CVD	849 (60.6)	635 (45.4)	846 (60.4)

© 2019 Nguyen CA et al. *JAMA Network Open.*

eTable 5. Regression Results for Process Measures

Variable	HbA_{1c} Testing (Diabetes)	LDL-C Testing (Diabetes)	LDL-C Testing (CVD)	Statin Use (Diabetes)	Statin Use (CVD)
Age (18-35, 36-45, 46-55, 56-65)	0.02***	0.03***	0.03***	0.12***	0.13***
Male	0.007***	0.01***	0.003***	0.08***	0.10***
Atrial fibrillation	-0.009**	0.0007	-0.02***	0.01	-0.01***
Hypertension	0.10***	0.12***	0.12***	0.12***	0.13***
COPD	-0.03***	-0.03***	-0.03***	-0.02**	-0.001
Heart failure	-0.04***	-0.04***	-0.05***	0.06***	0.06***
Chronic kidney disease	0.001	-0.01***	-0.02***	0.05***	0.06***
DxCG composite	-0.002***	-0.003***	-0.001***	-0.003***	-0.0007***
% Black	-0.0001***	-0.0002***	-0.00008***	-0.0006***	-0.0004***
% Hispanic/Latino	0.0001***	0.0003***	0.0003***	-0.00002	-0.00003
% College-educated	0.0003***	0.0003***	0.0003***	0.0002**	-0.00005
Geography (urban, suburban, rural)	-0.0009	-0.001	-0.001*	-0.002	-0.001
% Below poverty	-0.0006***	-0.001***	-0.0006***	-0.0005**	-0.0003***
Constant	0.77***	0.67***	0.64***	0.08***	-0.03***
R^2	0.05	0.06	0.04	0.10	0.11
Number of observations	729,483	729,483	2,933,947	348,048	1,341,536

*p<0.05, **p<0.01, ***p<0.001

eTable 6. Regression Results for Disease Control Measures

Variable	HbA_{1c} Level Control (Diabetes)	LDL-C Level Control (Diabetes)	LDL-C Level Control (CVD)
Age (18-35, 36-45, 46-55, 56-65)	0.06***	0.06***	0.05***
Male	-0.06***	0.08***	0.08***
Atrial fibrillation	0.04***	0.04***	0.05***
Hypertension	-0.005**	0.05***	0.10***
COPD	-0.006	-0.02**	0.004
Heart failure	-0.06***	0.03***	0.07***
Chronic kidney disease	-0.06***	0.03***	0.08***
DxCG composite	0.001***	0.0002	0.004***
% Black	-0.0006***	-0.0008***	-0.0005***
% Hispanic/Latino	-0.0005***	-0.0001	0.0001*
% College-educated	0.0008***	0.0004	-0.0003
Geography (urban, suburban, rural)	-0.004	-0.002	-0.005***
% Below poverty	-0.001***	-0.0006***	-0.0003**
Constant	0.55***	0.33***	0.16***
R^2	0.04	0.04	0.04
Number of observations	349,207	341,769	1,279,728

*p<0.05, **p<0.01, ***p<0.001

© 2019 Nguyen CA et al. *JAMA Network Open.*

eTable 7. Regression Results for Use-Based Outcome Measures

Variable	Hospital Admissions (Diabetes)	Hospital Admissions (CVD)
Age (18-35, 36-45, 46-55, 56-65)	0.02***	-0.0001*
Male	0.001*	-0.006***
Atrial fibrillation	-0.06***	-0.03***
Hypertension	-0.06***	-0.01***
COPD	-0.08***	-0.01***
Heart failure	-0.15***	-0.07***
Chronic kidney disease	-0.02***	0.02***
DxCG composite	-0.01***	-0.005***
% Black	-0.0003***	0.00002***
% Hispanic/Latino	0.00002	0.00001*
% College-educated	0.0002***	0.00002***
Geography (urban, suburban, rural)	0.001	0.00006
% Below poverty	-0.0007***	-0.00004**
Constant	0.92***	1.01***
R^2	0.14	0.09
Number of observations	729,483	2,933,947

*p<0.05, **p<0.01, ***p<0.001

eReferences

1. Schnake-Mahl AS, Sommers BD. Health care in the suburbs: An analysis of suburban poverty and health care access. *Health Aff*. 2017;36(10):1777-1785. doi:10.1377/hlthaff.2017.0545.

2. U.S. Census Bureau. Census regions and divisions of the United States. 2010. https://www2.census.gov/geo/pdfs/maps-data/maps/reference/us_regdiv.pdf.

3. Agency for Healthcare Research and Quality. Prevention Quality Indicators Technical Specifications Updates - Version 6.0 (ICD-9). 2016.

© 2019 Nguyen CA et al. *JAMA Network Open.*
